# *Linc00662* sponges *miR-15b-5p* to promote hypopharyngeal squamous cell carcinoma progression by facilitating cancer stem cell-like phenotypes

**DOI:** 10.7150/jca.95852

**Published:** 2024-05-20

**Authors:** Binghuang Zhang, Qing Ye

**Affiliations:** Shengli Clinical Medical College of Fujian Medical University, Fuzhou 350001, China.

**Keywords:** hypopharyngeal squamous cell carcinoma, *Linc00662*, * miR-15b-5p*, cancer stem cell

## Abstract

**Background:** Long non-coding RNAs (lncRNAs) are associated with multiple head and neck tumors and play important roles in cancer. This study explored the molecular mechanism of *Linc00662* in hypopharyngeal squamous cell carcinoma (HSCC).

**Methods:** Real-time quantitative reverse transcription polymerase chain reaction (qRT-PCR) was used to detect gene expression in HSCC tissues. The viability and proliferation of tumor cells were measured using CCK-8 assays. HSCC cell apoptosis was measured using flow cytometry and western blotting. Cell stemness was examined using the sphere formation assay. A xenograft tumor model was established to investigate the role of *Linc00662 in vivo*.

**Results:** The expression level of *Linc00662* in HSCC tissues was significantly higher than that in adjacent normal tissues. The expression of *Linc00662* had no significant relationship with the tumor stage. Patients with high *Linc00662* expression were found to have shorter overall survival than those with low *Linc00662* expression. *Linc00662* over-expression promoted cell viability and inhibited apoptosis. Using online databases and a dual luciferase reporter, *miR-15b-5p* was confirmed as a potential downstream sponge of *Linc00662*. Moreover, *Linc00662* was negatively associated with *miR-15b-5p* in HSCC cells. Depletion of *miR-15b-5p* can reverse the function of *Linc00662 in vivo* and *in vitro*. Furthermore, *Linc00662* promotes tumor growth, which was abolished by *miR-15b-5p* mimics. Importantly, the stemness of cancer stem cells was mediated by the *Linc00662*/*miR-15b-5p* axis.

**Conclusion:** Patients with HSCC with high *Linc00662* showed poor prognosis and high *Linc00662* induced stemness of tumor cells by targeting *miR-15b-5p*. *Linc00662* may serve as a novel diagnostic and target marker for head and neck squamous cell carcinoma.

## Introduction

According to the 2020 Global Cancer Report, head and neck squamous cell carcinoma (HNSC) is the sixth most common cancer in the world, with approximately 700,000 cases diagnosed per year 1. However, hypopharyngeal squamous cell carcinoma (HSCC) has the worst prognosis among all head-and-neck cancers, accounting for 0.8-1.5% of malignant tumors of HNSC, because it is generally detected at an advanced stage due to a lack of biomarkers for early diagnosis 2-4. Treatment options for HSCC are limited, causing the incidence rate of HSCC to rise rapidly, and there has been a trend toward younger patients in recent years 3, 5, 6. In particular, the five-year overall survival (OS) rate of patients with HSCC treated with radical radiotherapy was 25-40%, while those who received radiotherapy or chemotherapy only was 12-14% 7-9. Therefore, it is very important to study the mechanism of the occurrence and development of HSCC and explore new therapeutic targets for improving the survival time and quality of life of patients with HSCC.

HSCC is associated with submucosal development and has a high risk of lymph node metastasis 10, 11. Cancer stem cells (CSCs) are a small subset of tumors with the ability to regenerate and differentiate, and are considered to be one of the main drivers of tumor cell growth, migration, drug resistance, cancer recurrence, and metastasis in HNSC 12, 13. Therefore, CSCs play a key role in the development and metastasis of HNSC, explaining the heterogeneity and resistance to cancer therapy in cancers, including head and neck cancers 14. Therefore, targeting CSCs may be a new and effective strategy for treating HSCC.

Long non-coding RNAs (lncRNAs) have been identified as candidate tumor biomarkers that are expressed in different tissues and cancer types 15, 16. Recent studies have demonstrated that lncRNAs play an essential role in cancer cell stemness in HSCC 17-19. *Linc00662* is a newly discovered lncRNA, which is associated with poor prognosis and radiation and chemotherapy resistance in patients with cancer 20, 21. Xenograft experiments verified that *Linc00662* promotes breast cancer tumor growth and cell stemness *in vivo* 22. In particular, lncRNAs act as sponges for miRNAs to influence the progression of cancer cell stemness 23-27. There is increasing research on the roles of lncRNAs and *miR-15b-5p* in the occurrence and development of cancer 28-30. However, the role and function of *Linc00662* and *miR-15b-5p* in HSCC and their effects on tumor cell stemness have not been completely elucidated.

This study aimed to investigate the effect of *Linc00662* on cancer stemness and the underlying mechanism by which *Linc00662* regulates cancer stemness in HSCC by sponging *miR-15b-5p*. Our results may lead to the development of novel therapeutic strategies targeting CSCs in HSCC.

## Materials and Methods

### Data collection and analysis

The differential *Linc00662* expression in normal tissues of human hypopharyngeal carcinoma and at different tumor stages in patients with HSCC was analyzed using Gene Expression Profiling Interactive Analysis (GEPIA). Kaplan-Meier curves and relapse-free survival were used to test the association of *Linc00662* with OS and relapse-free survival. The binding of *miR-15b-5p* to *Linc00662* was monitored using miRDB, the Encyclopedia of RNA Interactomes (ENCORI), and the LncRNASNP2 online databases. *MiR-15b-5p* levels in HSCC were determined using the ENCORI database.

### Individuals

Seventeen Patients with HSCC were enrolled in The First Affiliated Hospital of Xiamen University (Xiamen, China), and all specimens were pathologically proven to have HSCC. Basic clinical information of the clinical samples is shown in** Table 1**. The tumor and surrounding tissues were frozen with liquid nitrogen after resection, and the samples were immediately stored at -80°C. All experiments were approved by the Ethics Committee of the First Affiliated Hospital of Xiamen University (XMYY-2021KYSB289) and Shengli Clinical Medical College of Fujian Medical University. The patients were fully briefed on the course of the study prior to inclusion, and written informed consent was obtained from all enrolled patients.

### Cell culture, transfection, and treatment

Human oral epithelial keratinocytes (HOK) and human head and neck cancer cell lines (FaDu, SCC-4, SCC-9, Hep-2, and CAL-27) were obtained from the American Type Culture Collection (Manassas, USA) and cultured in DMEM. These cells were supplemented with 10% fetal bovine serum in a humidified atmosphere containing 5% CO_2_ at 37°C. shRNA targeting *Linc00662* (ccggGGCTGATCTCACCTTGTAATTggatccAATTACAAGGTGAGATCAGCCtttttg) was subcloned into the pSin vector. The *miR-15b-5p* mimics and corresponding negative control miRNA were purchased from GenePharma Co., Ltd. (Shanghai, China). The pcDNA 3.1-*LINC00662* overexpression plasmid and corresponding empty vector were obtained from RiboBio (Guangzhou, China). For cell transfection, the *Linc00662* overexpression plasmid or *Linc00662* shRNA was transfected into FaDu and SCC-9 cells for 48 h, or a miRNA inhibitor of *miR-15b-5p* mimic was transfected into FaDu cells for 48 h, as instructed by the manufacturer. After 48 h, the FaDu and SCC-9 cells were collected. The experiments were repeated thrice.

### Real‐time quantitative reverse transcription‐polymerase chain reaction (qRT-PCR)

Total RNA was extracted from HNSC and mouse tissues treated with different groups. cDNA was synthesized using the PrimeScript RT Reagent Kit (Takara, RR047A). Approximately 1 μg of RNA from tissue was reverse transcribed using the Prime-Script miRNA cDNA Synthesis Kit (TaKaRa) and qRT-PCR was performed to detect the expression levels of *Linc00662*, *miR-15b-5p*, *CD44, Sox2, Nanog*, and *Oct4* usinf a SYBR Green Premix Ex Taq kit (Takara RR820A) in triplicate using synthesized primers (Tsingke, China). GAPDH and U6 served as internal controls. Primers used are listed in **Table 2**. All PCR assays were repeated thrice.

### Cell counting kit-8 (CCK-8)

A CCK-8 assay was performed according to the manufacturer's protocol (Dojindo, Japan) to evaluate the proliferation of FaDu and SCC-9 cells. Both cell types were seeded into 96-well plates at a density of 1 × 10^3^ cells/well. DMEM containing 10% CCK8 solution was added to each well and incubated for 24, 48, 72, and 96 h in a 37°C incubator. The absorbance of each well was measured at 450 nm using a microplate reader (Thermo Fisher Scientific, USA) to assess the efficiency of cell proliferation.

### Flow cytometry

An Annexin V-allophycocyanin apoptosis detection kit (eBioscience, San Diego, CA, USA) was used to detect apoptosis. In brief, FaDu and SCC-9 cells (1 × 10^6^ cells/mL) were stained with 5 μL Annexin V-FITC for 15 min and 10 μL PI in a staining buffer at 4°C with light avoidance for 5 min after being subjected to different treatments with phosphate-buffered saline (PBS), followed by flow cytometry (BD Biosciences, San Diego, CA, USA). For the detection of CD133 positive cells, cells were collected with a scraper, blocked with 3% BSA, and subsequently incubated with a CD133 antibody (CL488-66666, Proteintech) for 30 min on ice in staining buffer, according to the manufacturer's protocol. The data were analyzed three times and the mean value was calculated.

### Western blotting

Proteins (20 μg) were electro-transferred onto polyvinylidene fluoride (PVDF) membranes (Millipore, Darmstadt, Germany) after separation by 10% sodium dodecyl sulfate-polyacrylamide gel electrophoresis using a semi-dry blotting apparatus (Bio-Rad, Hercules, California, USA) and then PVDF were blocked with 5% nonfat milk at room temperature for 1 h and incubated with primary antibodies Bcl-2 (ab32124, Abcam, UK) and BAX (ab32503, Abcam) overnight at 4°C. On the second day, the membranes were incubated with appropriate secondary antibodies for 90 min and developed with enhancement. The protein bands were visualized using enhanced chemiluminescence (ECL Plus). The gray values of the protein bands were analyzed using ImageJ (National Institutes of Health, Bethesda, Maryland, USA). The experiment was repeated three times and the mean value was calculated.

### miRNA screening

Through miRDB, ENCORI, and LncRNASNP2 databases screening, candidate miRNAs were identified, and subsequently, these miRNAs were analyzed and compared based on the ENCORI database.

### Double luciferase reporter assay

Dual-luciferase reporter gene assay was performed to confirm the relationship between *miR-15b-5p* and *Linc00662*. Well-grown 293T cells (8 x10^4^ per well in a 24-well plate) were co-transfected with mutated *Linc00662* (*Linc00662*-MUT) and wild-type *Linc00662* (*Linc00662*-WT) together with the *miR-15b-5p* mimic or negative control (NC) using Lipofectamine 2000 according to the manufacturer's protocol (Thermo Fisher Scientific, USA). After transfection for 48 h, cells were collected and luciferase activity was measured using the dual-luciferase reporter assay system (Promega). All assays were performed independently in triplicate.

### Xenograft

The BALB/c nude mice (six weeks old, n=6/7) were purchased from Xiamen University and reared in plastic cages at 22-25 °C and a humidity of 40-70% under a 12 h/12 h light-dark cycle, with free access to water and food. After an adaptation period of one week, the mice were randomly divided into the following four groups: control, *Linc00662* model, *Linc00662*+NC mimic, and *Linc00662*+*miR-15b-5p* mimic. Tumor volume was measured every three d after the transfection of FaDu cells (2×10^7^) with *Linc00662* or *Linc00662* + *miR-15b-5p* mimic and their NC. Tumor volume was calculated using the following formula:

Tumor volume = 0.5×length×width^2^

On day 22 after inoculation, the mice were euthanized by CO_2_ inhalation (CO_2_ flow rate: 10% of cage volume), and the death of the animals was confirmed by the cessation of the heartbeat. The tumor xenografts were harvested, photographed, and weighed. The experiments were carried out in accordance with the protocol approved by the Animal Welfare Ethics Committee of The First Affiliated Hospital of Xiamen University (SYXK (Min)2018-0009).

### Tumorsphere assay

The protocol for the formation of tumorspheres has been described previously 31. Briefly, FaDu cells (5000 cells/well) with *Linc00662* or *Linc00662* + *miR-15b-5p* and a relative NC were seeded on six-well ultra-low attachment plates (Corning, New York, NY, USA) and cultured in DMEM/F12 without serum, with 2% B27, EGF 20 ng/mL, and FGF 20 ng/mL. The tumor spheres were recorded and counted on the 14th day after seeding using a microscope, and the number and size of the tumor spheres were analyzed.

### Statistical analysis

All data are presented as mean ± SD from at least three independent experiments. Statistical analyses were performed using IBM SPSS version 18.0. Student's t-test (unpaired and two-tailed) or one-way ANOVA was used to measure differences between two or more groups. The relationship between *Linc00662* and *miR-15b-5p* was measured using Spearman's rank test. Differences were considered statistically significant at P < 0.05.

## Results

### *Linc00662* is highly expressed in HSCC

**Figure 1:**
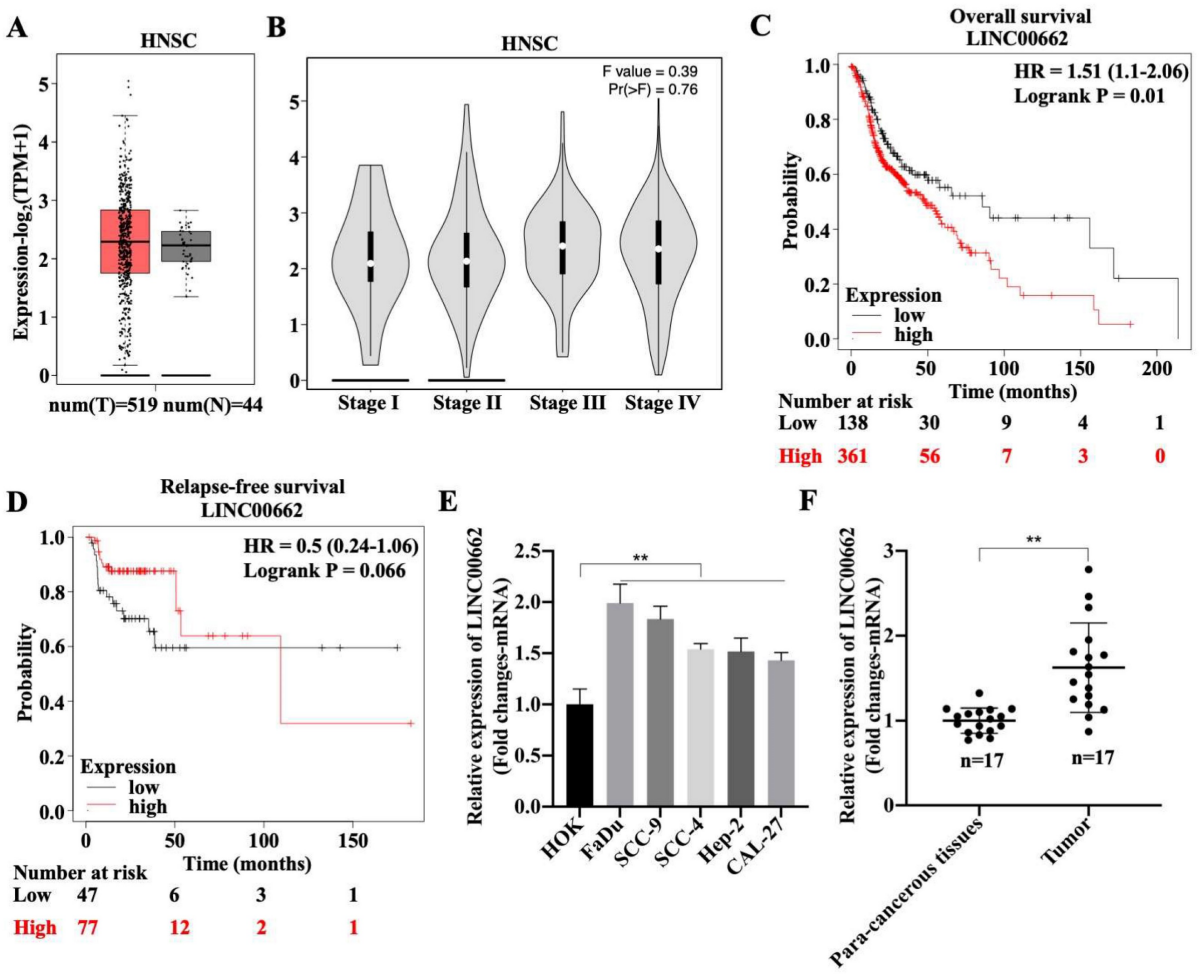
Expression and clinical value of lncRNA *Linc00662* in head and neck squamous cell carcinoma (HNSC). (A) Differential expressions of *Linc00662* in HNSC (n = 519) and not HNSC (n = 44) specimens were determined by using the GEPIA2 database. (B) The correlation between *Linc00662* and tumor stage in patients with HNSC expression. (C, D) The Kaplan-Meier method was used to determine the relationship between *Linc00662* expression and overall survival (C) or Relapse-free survival (D) of patients with HNSC. Differences between survival curves were tested by the log-rank test. Data are shown as the mean ± SD. Assays were performed in triplicate. (E) qRT-PCR analysis for *Linc00662* level in normal HOK and HNSC cell lines (FaDu, SCC-9, SCC-4, Hep-2, and CAL-27). (F) The expression of *Linc00662* in para-carcinoma and hypopharyngeal squamous cell carcinoma (HSCC) tissues. **P < 0.01.

To explore the role of *Linc00662* in the development of HSCC, the expression pattern of *Linc00662* was determined using the online database, GEPIA2. There was no differential expression of *Linc00662* between healthy individuals (n = 44) and patients with HNSC (n = 519), but *Linc00662* was highly expressed in patients with HNSC compared with that in healthy individuals (Fig. 1A). Although we found that *Linc00662* expression was not correlated with tumor stage or relapse-free survival in patients with HNSC (Fig. 1B-D), patients with high *Linc00662* expression survived shorter than those with low *Linc00662* expression (Fig. 1C). Subsequently, FaDu, SCC-9, SCC-4, Hep-2, and CAL-27 cells and normal head and neck HOK cells were cultured; the level of *Linc00662* was substantially higher in HNSC cell lines than that in normal head and neck cells. There were more *Linc00662* levels in FaDu and SCC-9 cells than the other two cell lines, which implied that *Linc00662* may have important roles in HSCC (Fig. 1E). Additionally, *Linc00662* was highly expressed in fresh HSCC tissues (n = 17), in contrast to the corresponding para-carcinoma tissues (Fig. 1F). Taken together, these results indicated that *Linc00662* may be involved in HSCC progression.

### *Linc00662* promotes cell activity and inhibits cell apoptosis in FaDu and SCC-9 cells

**Figure 2:**
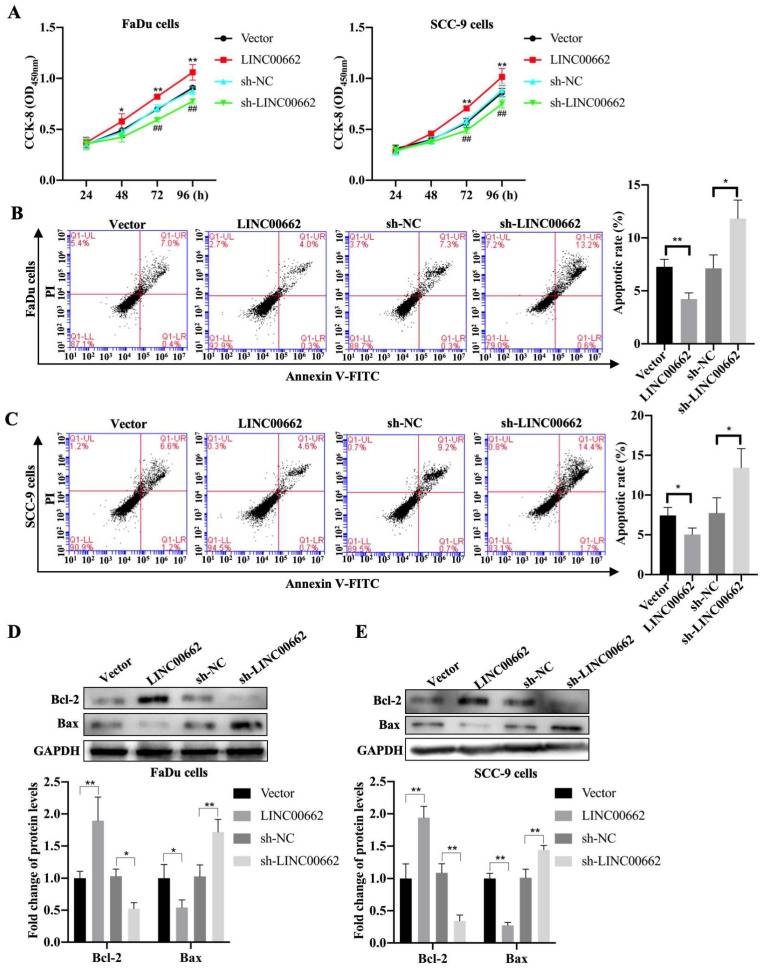
***Linc00662* affects the proliferation and apoptosis of** FaDu and SCC-9 cells. FaDu cells were transfected with si-*Linc00662* or *Linc00662* and control groups. (A, B) CCK-8 assay was used to test proliferation of FaDu (A) and SCC-9 cells (B) at 24, 48, 72, and 96 h, respectively. (C, D) Flow cytometry shows the apoptotic rate in FaDu (C) and SCC-9 cells (D) in *Linc00662* overexpression, si-*Linc00662*, and their control groups, respectively. (E, F) Western blot analysis was conducted to detect the expression levels of Bcl-2 and Bax in FaDu (E) and SCC-9 cells (F). All data were expressed as the mean ± SD, *P < 0.05, **P < 0.01.

To further investigate whether *Linc00662* impacted cell proliferation, we performed CCK-8 assays to detect the viability of FaDu and SCC-9 cells after *Linc00662* transfection. *Linc00662* significantly prompted the growth rate of FaDu and SCC-9 cells at 72 and 96 h compared to that in the control group. Knockdown of *Linc00662* contributed to a conspicuous reduction in cell proliferation in FaDu and SCC-9 cells compared with that in cells transfected with scrambled shRNA (Fig. 2A, B). Flow cytometry analysis suggested that *Linc00662* markedly reduced cell apoptosis and *Linc00662* silencing notably repressed apoptosis in FaDu and SCC-9 cells (Fig. 2C, D). Additionally, western blot analysis showed that Bcl-2 levels increased and Bax levels decreased in the *Linc00662*-overexpressing group while *Linc00662* silencing caused a reduction in Bcl-2 levels and increased Bax levels (Fig. 2E, F). In summary, our data reveal that *Linc00662* is a regulatory factor that promotes cell proliferation and inhibits apoptosis in HSCC cells.

### *Linc00662* sponges miR-15b-5p in HSCC

**Figure 3:**
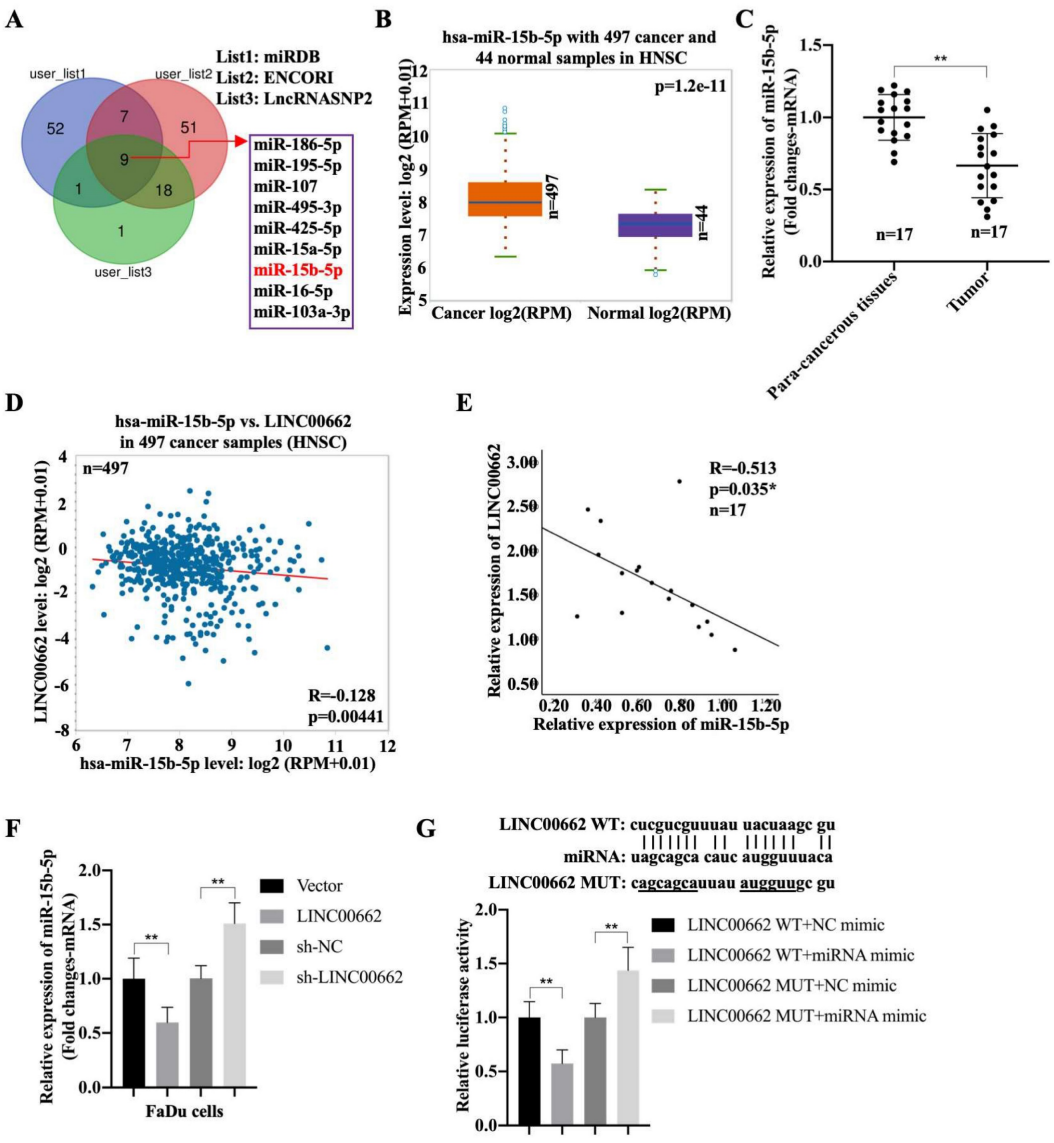
***Linc00662* directly binds to *miR-15b-5p* in FaDu and 293T cells (A)** The candidate miRNAs which could potentially target *Linc00662* were listed according to miRDB, ENCORI and LncRNASNP2.** (B)** Expression of *miR-15b-5p* in 497 patients with HNSC and 44 normal samples. **(C)** The expression of *miR-15b-5p* in para-carcinoma and HSCC cancer tissues. **(D)** The association between *miR-15b-5p* and *Linc00662* in HNSC was analyzed using ENCORI Starbase. **(E)** The association between *miR-15b-5p* and *Linc00662* in HSCC tissues. **(F)** The expression of *miR-15b-5p* mRNA were assessed by qRT-PCR in FaDu cells. **(G)** Luciferase reporter assay was performed to verify the interaction of *miR-15b-5p* and *Linc00662* in 293T cells. **P < 0.01.

To better understand the mechanism of *Linc00662* in regulating the progression of HSCC, night candidate miRNAs, including hsa-*miR-15b-5p*, were identified using the miRDB, ENCORI, and LncRNASNP2 databases (Fig. 3A). The results showed that the negative correlation between *miR-16-5p or miR-107* with *LINC00662* in patients with HNSC, but both associations were lower than the association between *miR-15b-5p* with *LINC00662*. Moreover, high expression of *miRNA-15b* was associated with poor prognosis, whereas low expression was associated with good prognosis (Fig. S1). Therefore, we selected *miR-15b-5p* as a candidate gene. In addition, *miR-15b-5p* was significantly expressed in patients with HNSC compared to that in the normal groups (Fig. 3B). Furthermore, *miR-15b-5p* was highly expressed in fresh HSCC tissues than that in para-carcinoma tissues (Fig. 3C). *MiR-15b-5p* also appeared to be negatively associated with *Linc00662* in HNSC, and a significant negative correlation was observed between *miR-15b-5p* and *Linc00662* in patients with HSCC (Fig. 3D, E). FaDu cells were transfected with *Linc00662* and a combination of *Linc00662* and *miR-15b-5p* mimic. qRT-PCR assays illustrated that *Linc00662* silencing increased *miR-15b-5p* expression, whereas *Linc00662* overexpression decreased *miR-15b-5p* expression in FaDu cells (Fig. 3F). Additionally, the relative luciferase activity of the *Linc00662*-WT reporter, rather than *Linc00662*-MUT reporter, was decreased by *miR-15b-5p* overexpression (Fig. 3G). These data confirm the direct binding of *miR-15b-5p* to *Linc00662* in HSCC.

### *Linc00662* promotes cell activity and inhibits cell apoptosis through sponging *miR-15b-5p*

**Figure 4:**
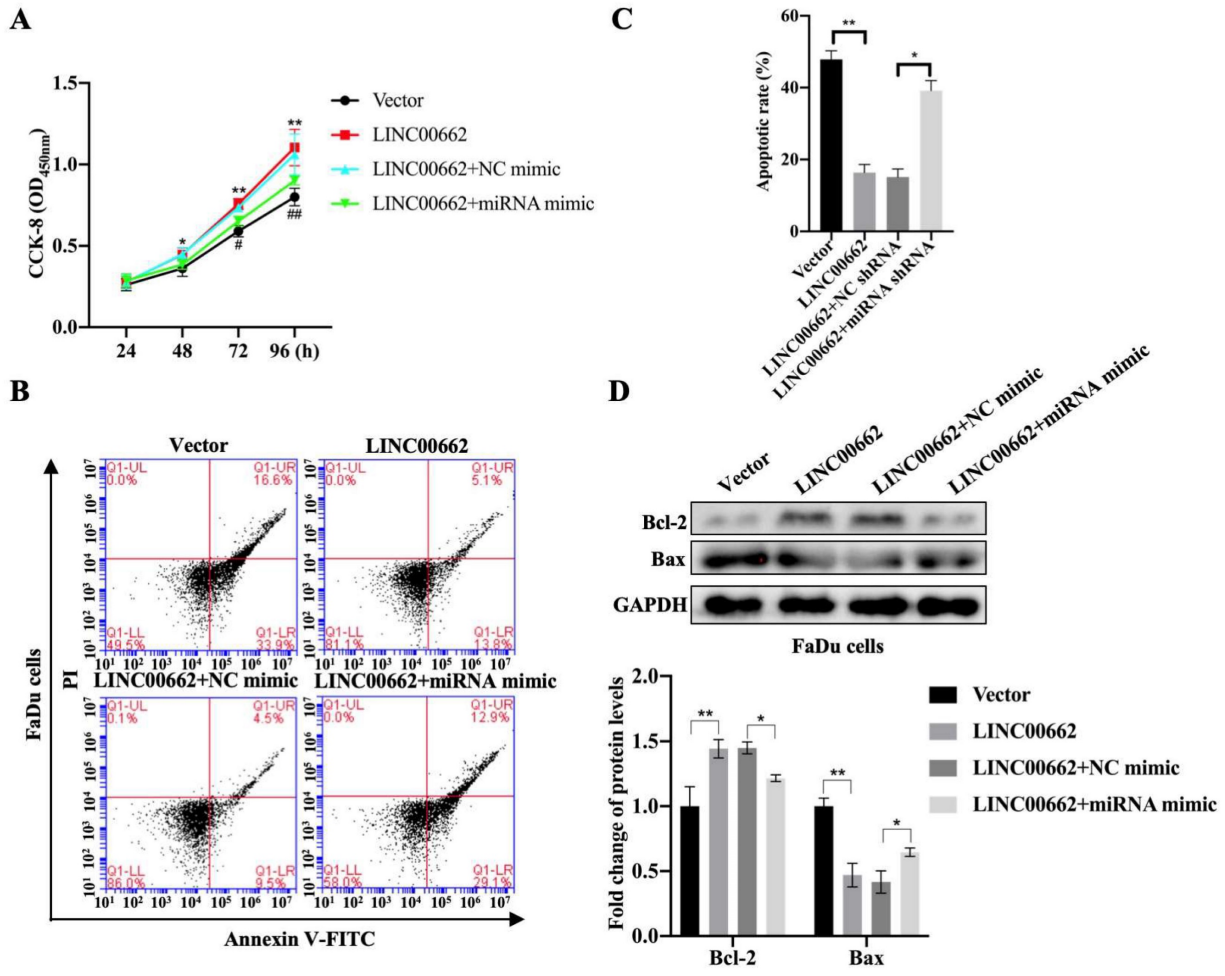
***Linc00662* can suppress *miR-15b-5p* expression.** FaDu cells were transfected with *Linc00662*, *Linc00662* + *miR-15b-5p* mimic or their corresponding control. **(A)** Cell proliferation was determined with the CCK-8 assay at the indicated time-points.** (B)** Flow cytometric analysis of the cell cycle in FaDu cells stained with propidium iodide. **(C)** The apoptotic results were evaluated by flow cytometry. **(D)** Apoptosis-marker Bcl-2 and Bax were determined by western blotting in FaDu cells. *P<0.05, **P<0.01.

To determine whether *Linc00662* could regulate the progression of HSCC by targeting *miR-15b-5p*, FaDu cells were transfected with *Linc00662* alone or a combination of *Linc00662* and *miR-15b-5p* mimic. The CCK-8 assay showed that *Linc00662* overexpression significantly promoted FaDu cell proliferation compared with that in the control, whereas the *miR-15b-5p* mimic reversed the effect of *Linc00662* (Fig. 4A). Flow cytometry analysis further demonstrated that overexpression of *Linc00662* inhibited apoptosis. However, after transfection with the *miR-15b-5p* mimic, the effect of *Linc00662* on cell apoptosis was significantly reversed (Fig. 4B, C). Furthermore, *Linc00662* overexpression induced an increase in Bcl-2, which was prevented by treatment with the *miR-15b-5p* mimic, whereas the pro-apoptotic Bax protein exhibited the opposite results (Fig. 4D). These data demonstrated that the *Linc00662*/*miR-15b-5p* axis can decelerate HSCC progression.

### The *Linc00662*/*miR-15b-5p* pathway promotes tumor growth of HSCC

**Figure 5:**
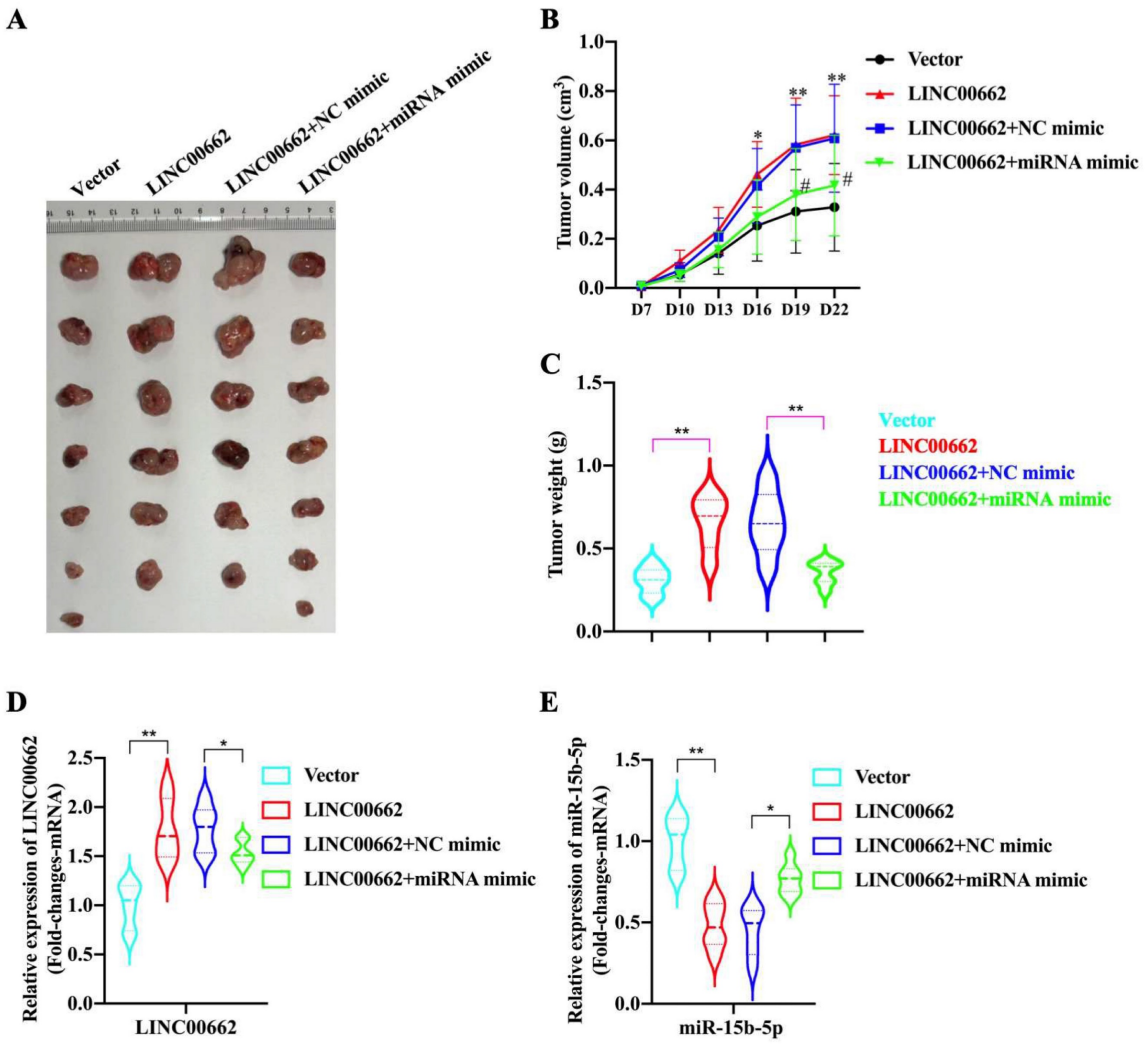
***Linc00662*/*miR-15b-5p* modulates HSCC growth in vivo. (A)** Representative images of tumor tissues from the FaDu cells transfected with *Linc00662* or the combination of *Linc00662* and *miR-15b-5p* mimic for 72 h were obtained on day 7. Vector (n=7): FaDu cell transfected with the vector; *LINC00662* (n=6): FaDu cell transfected with *LINC00662* overexpression plasmid; *LINC00662*+NC mimic (n=6): FaDu cell transfected with *LINC00662* overexpression plasmid with negative control mimic; *LINC00662*+miRNA mimic (n=7): FaDu cell transfected with *LINC00662* overexpression plasmid with *miR-15b-5p* mimic. **(B)** Tumor volume was calculated every 3 d after injection. **(C)** Weight of the mouse body was measured at the end of modeling. **(D)** The expression level of Linc000662 in tumor tissue as determined using qRT-PCR. **(E)** The expression level of *miR-15b-5p* in tumor tissue as determined using qRT-PCR. *P < 0.05, **P < 0.01.

In the tumor growth xenograft model, FaDu cells were subcutaneously implanted into nude mice to induce tumor development (n = 6/7 for each group). After 22 d of injection, the mice were sacrificed, and tumors were obtained (Fig. 5A). We found that the promoting effect of *Linc00662* on tumor volume was significantly reversed after transfection with *miR-15b-5p* mimics compared with that in the control group (Fig. 5B, C). Additionally, the weight of tumors transfected with *Linc00662* significantly increased compared with that of the control group; however, when co-transfected with *Linc00662* and *miR-15b-5p* mimic, the stimulatory effect induced by *Linc00662* was rescued (Fig. 5C). The qRT-PCR results further suggested that the *miR-15b-5p* mimic transfection group can significantly reduce the expression of *Linc00662* compared with that in the control group (Fig. 5D). Similarly, the expression of *miR-15b-5p* in the *miR-15b-5p* mimic transfection group was significantly higher than that in the *LINC000662* overexpression alone (Fig. 5E). Overall, these data indicate that *Linc00662* promotes HSCC growth in the xenograft model, while the *miR-15b-5p* mimic could rescue this result.

### Cancer cell stemness induced by *Linc00662* in HSCC cells is dependent on *miR-15b-5p*

**Figure 6:**
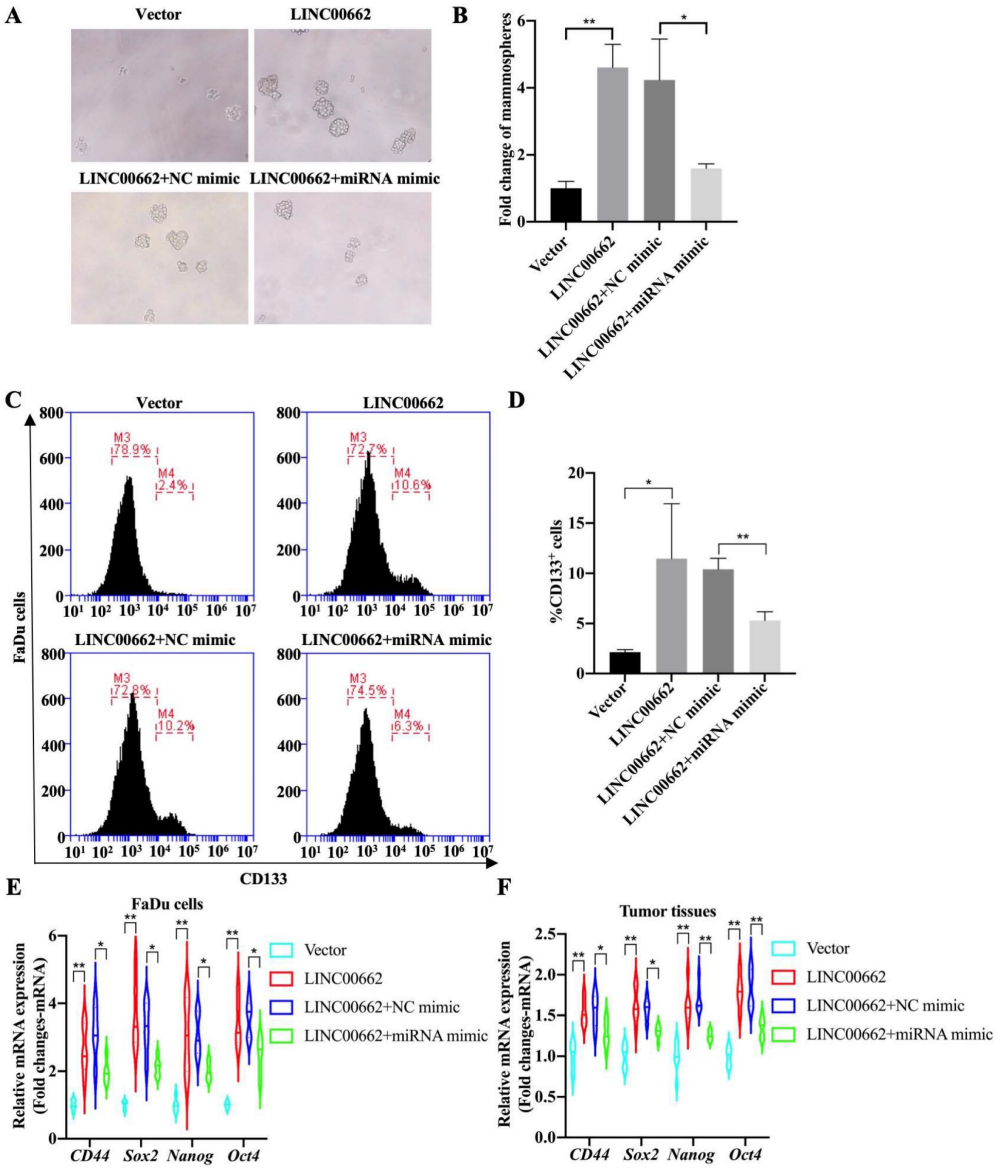
***Linc00662*/*miR-15b-5p* regulates cancer stem cell properties in HSCC. (A)** Tumorsphere formation assays of *Linc00662*, *Linc00662* + *miR-15b-5p* mimic or their corresponding control **(B)** Quantitation analysis of tumorsphere assay. **(C)** Flow cytometry analysis shows the proportion of CD133-positive cells in the FaDu cells co-transfected with *Linc00662* and *miR-15b-5p* mimic and the corresponding controls.** (D)** Quantitative result for CD133-positive cell proportion. **(E, F)** The expression of *CD44, Sox2, Nanog,* and *Oct4* in FaDu cells and tumor tissues was determined by qRT-PCR. *P < 0.05, **P < 0.01.

Tumor cell stemness is one of the main factors determining the progression of HSCC, therefore, whether the *Linc00662*/*miR-15b-5p* axis regulates the stemness of pharyngeal cancer cells was further explored and tumorsphere assays were evaluated. The results suggested that overexpression of *Linc00662* promotes pheroidization of FaDu cells, but transfection with *miR-15b-5p* mimics significantly reversed the *Linc00662*-mediated promoting effect of cancer cell stemness (Fig. 5A, B). Flow cytometry analysis showed that *Linc00662* increased the proportion of CD133-positive cells, and *miR-15b-5p* mimic transfection significantly abolished the promotive effects of *Linc00662* (Fig. 5C, D). Next, the expression of other CSC markers was detected in FaDu cells. qRT-PCR analysis showed that the relative mRNA expression of *CD44, Sox2, Nanog*, and *Oct4* increased after treatment with *Linc00662* compared to those in the control group, which was reversed by the *miR-15b-5p* mimic (Fig. 6E). In addition, CSC markers in tumor tissues in vivo showed similar results (Fig. 6F). Taken together, these results indicate that *Linc00662*/*miR-15b-5p* mediates the development of HSCC tumors by influencing tumor stem cells.

## Discussion

CSCs can initiate tumorigenesis and exist in many different tumor types. Targeting CSCs by suppressing unique molecular determinants of CSCs provides effective therapeutic intervention 32. However, the limited knowledge of CSCs in HSCC may provide new clinical targets for metastatic or recurrent HSCC. The recurrence and metastasis of HSCC may also be attributed to the persistent presence of CSCs. In this study, we proved that *Linc00662* is expressed in HSCC and could regulate the proliferation and apoptosis of HSCC cells by sponging *miR-15b-5p*, which in turn facilitates the appearance of a CSC-like phenotype. Therefore, this study provides an experimental and theoretical basis for the treatment of HSCC.

*Linc00662* is upregulated in malignant tumors 20, such as cervical cancer 33, gastric cancer 34, and osteosarcoma 35, which is also closely related to poor prognosis and chemotherapy resistance in patients with cancer 36, 37. In this study, we revealed that *Linc00662* expression is higher in patients with HNSC, and predicted poor prognosis and shorter survival. Notably, the expression of *Linc00662* was significantly higher in HSCC tissues and FaDu/SCC-9 cells. Possibly, *Linc00662* may play an important role in the development and occurrence of HSCC. *Linc00662* upregulation can affect the proliferation, invasion, and apoptosis of cervical cancer cells 38. In addition, overexpression of *Linc00662* facilitates the proliferation, migration, and invasion of oral squamous cell carcinoma cells 39 and promotes cell viability and metastasis in esophageal squamous cell carcinoma 40. Our data showed that highly expressed *Linc00662* promoted proliferation and inhibited apoptosis in HSCC cells. In contrast, knockdown of *Linc00662* restrained proliferation and promoted apoptosis. Therefore, *Linc00662* may be a positive regulator of HSCC development.

*MiR-15b-5p* has carcinogenic or tumor-inhibitory functions in different types of cancer, and its expression is upregulated in liver and breast cancers, and downregulated in castration-resistant prostate cancer cells 41-43. Low expression of *miR-15b-5p* is also associated with poor prognosis in patients with HCC 28 and *miR-15b-5p* can predict local recurrence in patients with head and neck cancer treated with intensity-modulated radiotherapy 44. In our data, the expression of *miR-15b-5p* was significantly lower in patients with HSCC than that in the normal population. Previous studies have indicated that the lncRNA *TRPM2-AS/miR-15b-5p/PPM1D* axis can promote malignant tumors in osteosarcoma cells 45 or *Linc00662* can act as a sponge for *miR-15b-5p*, facilitating the progression of osteoarthritis 46. Additionally, lncRNA-MEG8 accelerates non-small cell lung cancer progression by regulating the *miR-15b-5p*/PSAT1 axis 47. In this study, our results showed that the *Linc00662*/*miR-15b-5p* axis affects HSCC cells and tumors in vivo. There is evidence indicating that the lncRNA *FENDRR/miR-15b-5p/TUBA1A* axis suppresses cervical cancer cell proliferation and invasion 48, and the lncRNA *TTN-AS1* suppresses ovarian cancer cell proliferation and invasion by targeting *miR-15b-5p* and regulating FBXW7 expression 49. Therefore, these studies indicate that *miR-15b-5p* exhibits substantial potential in regulating tumor cell proliferation and tumor growth. In our study, we proved that *Linc00662* promoted the proliferation and inhibited the apoptosis of HSCC cells, which was reversed by the *miR-15b-5p* mimic. Therefore, activation of the *Linc00662*/*miR-15b-5p* signaling axis can accelerate the proliferation of HSCC cells, thereby leading to the growth of HSCC in vivo.

CSCs have a high degree of plasticity and are embedded in tumors, making them difficult to identify and eradicate 50, 51. They are usually identified by the expression of cell surface markers, such as CD133, which is one of the most well-characterized biomarkers used to isolate CSCs 52; CD44 is usually a highly expressed surface marker in CSC 53, and Sex2, Nanog, and Oct4 are transcription factors necessary for maintaining the phenotype of pluripotent embryonic stem cells 54, 55. CSCs in oral/oropharyngeal squamous cell carcinoma enhance the tumorigenicity and self-renewal abilities of cancer cells in vivo 56, 57. Moreover, many studies have reported that *Linc00662* enhances cell stemness in breast cancer/osteosarcoma by sponging *miR-144-3p/miR-16-5p* 22, 35, and that lncRNA *CERS6-AS1* acts as an oncogene that facilitates xenograft tumor growth by binding to *miR-15b-5p* 29. Consistent with previous findings, we demonstrate that the *Linc00662*/*miR-15b-5p* axis affects tumor cell growth stemness. Thus, the *Linc00662*/*miR-15b-5p* axis provides new insights into the targeted mechanisms in HSCC.

## Conclusion

In summary, *Linc00662* acts as an oncogene that promotes stemness of HSCC cells and facilitates xenograft tumor growth by binding to *miR-15b-5p*. These findings may shed new light on the underlying mechanisms and treatments for HSCC. However, this study has some limitations. First, a larger clinical sample size of hypopharyngeal cancer is still needed to confirm the findings of this study. Second, 3D cell culture technology needs to be employed to confirm the role of the *Linc00662*/*miR-15b-5p* axis in tumor cell stemness.

## Supplementary Material

Supplementary figure.

## Figures and Tables

**Table 1:** The basic clinical information of 17 cases squamous cell carcinoma patients.

Patient number	Differentiation degree	Tumor stage	Year	Gender	Lymph node metastasis
932859	Medium-low differentiation	T2N0M0	68	Male	Yes
930603	High-medium differentiation	T4N2M0	60	Male	No
931192	Moderately differentiation	T2N0M0	54	Male	No
931376	Moderately differentiation	T4aN2cM0	62	Male	No
922354	High-medium differentiation	T4aN1M0	65	Male	No
925424	Medium-low differentiation	T4aN1M0	57	Male	Yes
926619	Moderately differentiation	T3N1M0	59	Male	No
908026	Poorly differentiation	T4N0M0	54	Male	Yes
871489	Poorly differentiation	T4N0M0	50	Male	Yes
922673	Moderately differentiation	T3N2M0	53	Male	No
447883	High-medium differentiation	T4aN0M0	59	Male	No
933192	Moderately differentiation	T2N0M0	59	Male	No
933196	High-medium differentiation	T2N0M0	53	Male	No
930720	Medium-low differentiation	T4N0M0	56	Male	Yes
943755	Moderately differentiation	T3N0M0	66	Male	Yes
940629	High-medium differentiation	T4aN2bM0	62	Male	No
942006	Moderately differentiation	T4aN2bM0	59	Male	No

**Table 2:** Primer sets used for quantitative reverse transcription-PCR.

Gene	Forward primer	Reverse primer
***Linc00662***	5′-ACACGCTTCTGAAACTGG-3′	5′-GTCAACATGGTGAAACCC-3′
***Sox2***	5′-TGCACCGCTACGACGTGAGC-3′	5′-GCCCTGGAGTGGGAGGAAGA-3′
***CD44***	5′-TGAGCATCGGATTTGAGAC-3′	5′-CATACTGGGAGGTGTTGGA-3′
***Oct4***	5′-AACGATCAAGCAGTGACTATTC-3′	5′-GAGTACAGGGTGGTGAAGTGAGG-3′
***Nanog***	5′-AGTTGGACAGGGAGATGGC-3′	5′-AACCTTCCTTGCTTCCACG-3′
***GAPDH***	5′-ACAACTTTGGTATCGTGGAAGG-3′	5′-GCCATCACGCCACAGTTTC-3′
***miR-15b-5p***	5'-UAGCAGCACAUCAUGGUUUACA-3'	5'-CTCAACTGGTGTCGTGGA-3'
***U6***	55'-CTCGCTTCGGCAGCACAT-3'	5'-ACGCTTCACGAATTTGCGT-3'
***NC mimic***	5'-UUCUCCGAACGUGUCACGUTT-3'	5'-ACGUGACACGUUCGGAGAATT-3'
***miR-15b-5p mimic***	5'-UAGCAGCACAUCAUGGUUUACA-3'	5'-UAAACCAUGAUGUGCUGCUAUU-3'

## Abbreviations

HSCC: hypopharyngeal squamous cell carcinoma
HNSC: head and neck squamous cell carcinoma
qRT-PCR: Real‐time quantitative reverse transcription‐polymerase chain reaction
CSCs: Cancer stem cells
lncRNAs: Long non-coding RNAs
PVDF: polyvinylidene fluoride
SDS-PAGE: sodium dodecyl sulfate-polyacrylamide gel electrophoresis
FCM: Flow cytometry
