# Supplementary material for: Linc00662 sponges miR-15b-5p to promote hypopharyngeal squamous cell carcinoma progression by facilitating cancer stem cell-like phenotypes
Source: J Cancer. 2024 May 20;15(12):3781–93. doi: 10.7150/jca.95852 (PMC11190759; doi:10.7150/jca.95852)
Supplement: Supplementary file 1 — Supplementary figure. [file jcav15p3781s1.pdf]

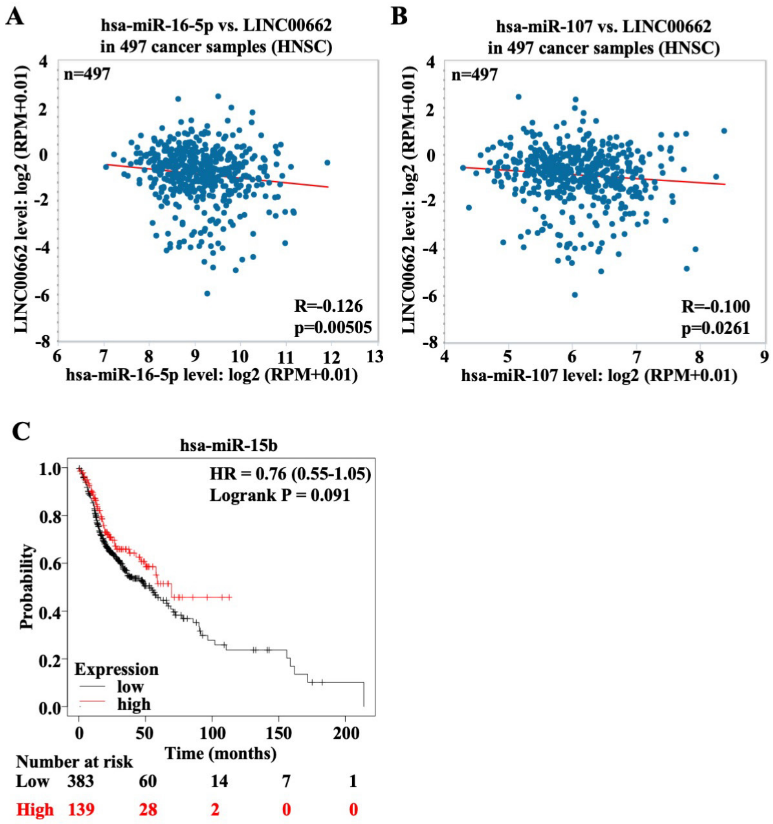

Figure S1 Correlation analysis of *Linc00662* and other targeted miRNAs. (A, B) The association between *miR-16-5p* (A) or *miR-107* (B) and *Linc00662* in HNSC as analyzed using ENCORI Starbase. **(C)** The Kaplan–Meier method was used to determine the relationship between *miR-15b-5p* expression and overall survival of patients with HNSC. Differences between survival curves were tested by the log-rank test. Data are shown as the mean ± SD. Assays were performed in triplicate.
